# Actual implementation of sick children’s rights in Italian pediatric units: a descriptive study based on nurses’ perceptions

**DOI:** 10.1186/s12910-015-0021-0

**Published:** 2015-05-13

**Authors:** Sofia Bisogni, Corinna Aringhieri, Kathleen McGreevy, Nicole Olivini, José Rafael Gonzalez Lopez, Daniele Ciofi, Alberta Marino Merlo, Paola Mariotti, Filippo Festini

**Affiliations:** Department of Health Sciences, University of Florence, Florence, Italy; Don Gnocchi Foundation, Florence, Italy; Meyer Children’s University Hospital of Florence, Florence, Italy; Department of Nursing, University of Sevilla, Seville, Spain; Health Authority of Florence, Torregalli Hospital, Florence, Italy

**Keywords:** Children, Hospital, Charter of rights

## Abstract

**Background:**

Several charters of rights have been issued in Europe to solemnly proclaim the rights of children during their hospital stay. However, notwithstanding such general declarations, the actual implementation of hospitalized children’s rights is unclear. The purpose of this study was to understand to which extent such rights, as established by the two main existing charters of rights, are actually implemented and respected in Italian pediatric hospitals and the pediatric units of Italian general hospitals, as perceived by the nurses working in them.

**Methods:**

Cross-sectional study. A 12-item online questionnaire was set up and an invitation was sent by email to Italian pediatric nurses using professional mailing lists and social networks. Responders were asked to score to what extent each right is respected in their hospital using a numeric scale from 1 (never) to 5 (always).

**Results:**

536 questionnaires were returned. The best implemented right is the right of children to have their mothers with them (mean score 4.47). The least respected one is the right of children to express their opinion about care (mean 3.01). Other rights considered were the right to play (4.29), the right to be informed (3.95), the right to the respect of privacy (3.75), the right to be hospitalized with peers (3.39), the right not to experience pain ever (3.41), and the right to school (3.07). According to the majority of nurses, the most important is the right to pain relief.

Significant differences in the implementation of rights were found between areas of Italy and between pediatric hospitals and pediatric units of general hospitals.

**Conclusion:**

According to the perception of pediatric nurses, the implementation of the rights of hospitalized children in Italian pediatrics units is still limited.

## Background

During the 20th century people and institutions gradually became aware that children are vulnerable, that they need protection and, above all, that they have inalienable human rights. In 1989 the UN Convention on the Rights of the Child established the right of children to be protected, supported, and respected, to participate and to have their dignity recognized [1].

These principles were also applied to health care settings [2], revolutionizing pediatric care in Western countries. Until the mid-20th century, pediatric care had largely neglected the emotional needs of sick and hospitalized children. Even if psychologists had already demonstrated that intrapsychic and relational dynamics in childhood could be responsible for pathological conditions in adulthood, there remained a strong belief that the child’s mind was immune from disorders other than those of a purely organic nature [3,4].

The first studies on the psychology of sick children showed that there are huge differences in the experiences and unconscious fantasies of adults and children related to disease. Hospitalized children would show signs of serious changes in mood, changes in the relationships with their parents and siblings, onset of eating and sleeping disorders, apathy, enuresis, regression, or, on the contrary, a pathological acceleration in behavioral maturation [5]. The idea that hospitalized children are at risk of some disorders was reinforced when the work of Bowlby and Robertson clearly showed the psycho-physical consequences of the child’s separation from the rest of the family following hospitalization [6]. At that time children were often hospitalized for long periods of time during which their parents were excluded from wards. This separation generated a trauma in children that consisted of three stages: protest, despair, and detachment [7-10].

In 1959 the UK government's "Platt Report" analyzed the critical aspects of pediatric hospitalization and gave specific recommendations on how to reduce the psychological distress of hospitalized children, as well as its consequences. These recommendations included the elimination of limits to visits from family members, the possibility for a parent to stay overnight with their child, and the presence of play areas [11]. These recommendations are considered the foundation for all the following declarations on the rights of children in the hospital: they initiated the gradual transformation of pediatric care in hospitals [12,13] towards a model that would provide for the inclusion of families in the care of hospitalized children [14], the separation between pediatric and adult wards [15,16], the creation of spaces dedicated to play and education [17,18], the provision of information suited to the child’s age and condition [19], and an adequate management of pain [20].

In Europe, after an attempt of the European Parliament to adopt a European charter for children in the hospital that would ensure that the primary needs of young patients be met [21], in 1988 a group of associations and charities drew up the first charter of the rights of sick children, also called the "Leiden Charter" or the "Charter of the European Association for Children in Hospital" (EACH). This charter lists a number of rights that are valid before, during and after hospitalization for all sick children regardless of their disease, age, disability, origin, social or cultural background [22,23]. These rights include, among others, accommodation and support to parents, informed participation in all decisions and the provision of care by adequately trained staff in pediatric wards. Since the 1990s, the main principles of the EACH charter have gradually been implemented and concrete actions have been taken to protect sick children [24].

In Italy, two specific charters have been drawn up – one by the *Associazione Bambini in Ospedale* (ABIO, Association for Children in Hospital) [25], which is the Italian translation of the EACH charter, and one by the *Associazione Ospedali Pediatrici Italiani* (AOPI, Association of Italian Pediatric Hospitals) [19,26]. These documents have very clearly established the rights of hospitalized children and the duties of the staff and structures in charge of their care. However, at present no data is available on the actual implementation of the charters and on the actual respect of the rights established by them in the various Italian pediatric settings.

The hospitalization of children and adolescents remains a remarkable social and health care phenomenon in Italy: more than 1,200,000 children are hospitalized every year, equal to 88‰ of the population aged 0 to 18. Even if this rate is lower than in previous years (124‰ in 2000 and 104‰ in 2004), it remains considerably higher than in other countries such as the United Kingdom and the United States [27].

Knowing to which extent the rights of hospitalized children established by the charters are actually respected is of paramount importance for planning measures aimed at guaranteeing the rights of sick children and their psycho-physical wellbeing in the hospital.

In pediatric hospitals, due to their close and continuous interaction with children and their families, nurses play a very important role in ensuring the psycho-physical wellbeing of sick children and in effectively guaranteeing their right to a comfortable, non-stressful stay in the hospital. On the other hand, due to their physical proximity to children and their families, nurses are privileged observers who can detect any logistical, organizational and structural problems that hinder the implementation of the rights established by the charters.

This study aimed to understand to which extent the sick children's rights established by the two main charters – EACH/ABIO and AOPI – are actually implemented and respected in Italian pediatric hospitals and Italian pediatric units of general hospitals, according to the perceptions of the nurses working in them. The secondary goal of the study was to identify possible variables associated with the level of implementation of these rights.

## Methods

### Cross-sectional survey

The data were collected by distributing a questionnaire to Italian nurses working in pediatric hospitals and the pediatric units of general hospitals.

In order to define the tool to be used we first reviewed the literature regarding hospitalized children's rights to find a questionnaire suitable for the goals of our study. Only two papers that include a questionnaire for assessing the respect of the rights of hospitalized children were found [2,26]. The study by Migone et al. included a 47-item questionnaire administered to children's parents; each question related to a single activity that, according to the authors, contributed to the implementation of one child right. The questions could be answered "yes", "no" or "I don't know" [2]. A publication by the International Network of Health Promoting Hospitals and Health Services reported the results of a study regarding the actual implementation of hospitalized children's rights in 15 hospitals. The 12-item questionnaire was administered to the 15 managers of the hospitals, who were asked to self-evaluate to what extent each right from a list of 12 had been achieved in their hospitals. The questions could be answered "completely unconsidered", "slightly considered", "meaningfully considered" or "significantly achieved" [26]. We decided to adopt the latter tool as the basis to develop our questionnaire because the answers in the study by Migone seemed to return too little information due to the yes-no responses.

We then began informal discussions with a limited number of expert nurses from our hospital, selected from among those with the longest experience in the care of children, in order to collect expert opinions and proposals on how to adapt the chosen tool to the purpose of our study. Subsequently, we conducted focus groups of senior pediatric nurses where the opinions and suggestions previously collected were discussed. Since the questionnaire aimed to understand the implementation of children's rights indirectly, by means of nurses' perceptions, the focus group concluded to exclude from the investigation the implementation of those rights that nurses could not have direct knowledge of such as "Children shall be admitted to hospital only if the care they require cannot be equally well provided at home" [25] or "The adolescent has the right to have an unmediated and confidential contact with the physician" [19]. Moreover, the focus group established a univocal formulation for those rights that are defined in different ways in the two charters. Table 1 shows a synopsis of the two above-mentioned charters of children's rights in hospital. On the basis of the conclusions of the focus groups, we set up a 12-item questionnaire made of 10 multiple-choice questions, in which respondents had to choose a numerical value indicating the frequency with which a right is implemented in their hospital (1 = never, 2 = rarely, 3 = sometimes, 4 = often, 5 = always), plus 2 open-ended questions.

**Table 1:** **Synoptic table of the EACH and AOPI charters of sick children's rights**

**BIO/EACH charter**	**AOPI charter**
1. Children shall be admitted to hospital only if the care they require cannot be equally well provided at home or on a day basis.	1. Children have the right to the highest level of health possible.
2. Children in hospital shall have the right to have their parents or parent substitute with them at all times.	2. Children have the right to be cared for as a whole.
3. (1) Accommodation should be offered to all parents and they should be helped and encouraged to stay. (2) Parents should not need to incur additional costs or suffer loss of income (3) In order to share in the care of their child, parents should be kept informed about ward routine and their active participation encouraged.	3. Children have the right to receive the highest level of treatment and care.
4. (1) Children and parents shall have the right to be informed in a manner appropriate to age and understanding. (2) Steps should be taken to mitigate physical and emotional stress	4. Children have the right to respect for their personal, cultural and religious identity.
5. (1) Children and parents have the right to informed participation in all decisions involving their health care (2) Every child shall be protected from unnecessary medical treatment and investigation.	5. Children have the right to have their privacy guaranteed.
6. (1) Children shall be cared for together with children who have the same developmental needs and shall not be admitted to adult wards.(2) There should be no age restrictions for visitors to children in hospital.	6. Children have the right to the safeguarding of their own physical, psychic and relational development. They have the right to keep their relationships with others, even during isolation. Children also have the right not to be subjected to any form of restraint.
7. Children shall have full opportunity for play, recreation and education suited to their age and condition and shall be in an environment designed, furnished, staffed and equipped to meet their needs.	7. Children have the right to be informed about their health conditions and about the procedures they will undergo, according to their developmental stage and self-awareness level. They have the right to freely express their opinions on any matter and to have them taken into consideration.
8. Children shall be cared for by staff whose training and skills enable them to respond to the physical, emotional and developmental needs of children and families.	8. Children have the right to express their assent or dissent regarding decisions involving their health care.
9. Continuity of care should be ensured by the team caring for children.	9. Children have the right to express their assent or dissent regarding participation in clinical trials.
10. Children shall be treated with tact and understanding and their privacy shall be respected at all times.	10. Children have the right to express their discomfort or suffering as well as to undergo the least invasive and painful treatments.
	11. Children have the right to be protected against any form of maltreatment, abuse, violence or neglect.
	12. Children have the right to be trained to be independent as much as possible in the management of their own disease.
	13. Minors have the right to access healthcare professionals and ask them questions confidentially and without the intervention of their parents.
	14. Children and their families have the right to participation.

The questionnaire was administered to 10 nurses to test acceptability and comprehensibility. To test reliability, the questionnaire was administered to 50 nurses at our hospital: Cronbach’s alpha for internal consistency was then performed on the answers to the 10 closed-ended questions with a result of 0.81.

The questionnaires were distributed in paper form during congresses or training initiatives for pediatric nurses as well as by means of an online form, linked to a dedicated Facebook page.

The questionnaires were collected in the period from September 2012 to April 2013.

The questions concerned the rights established by the two Italian charters of the rights of sick children that have the biggest impact on the organization of pediatric care.

In the questionnaire, the responding nurses were asked how often the following children’s rights were truly guaranteed in their work settings:

- right to have their parents (or parent substitute) with them at all times;
- right to have proper and comfortable accommodation for their parents, in order to allow them to stay with their children during the whole day;
- right to the involvement and active participation of parents in their health care;
- right to play freely and to have their toys with them;
- right to receive information on their condition and on the procedures they will undergo in a manner appropriate to their age and understanding;
- right to express their informed assent or dissent with regard to diagnostic and therapeutic decisions involving them;
- right to be cared for together with children who have reached the same developmental stage;
- right to have their privacy respected at all times;
- right to continuity of education;
- right to never suffer from pain.

In the open-ended questions, respondents were asked which right they considered to be the most important for hospitalized children and which right was most frequently neglected. In this case, they were free to indicate any right, even if not included in the list above or in one of the charters.

Every respondent was also asked which region they came from and if they worked in a pediatric hospital or in the pediatric unit of a general hospital.

The collected data were anonymous and entered into an Excel spreadsheet.

For each question we calculated the mean scores of answers, as well as the percent frequency distribution of the possible answers.

The answers were stratified by area of origin (with a distinction being made between structures located in northern and central Italy and structures located in the south) and by setting (with a distinction between pediatric hospitals and pediatric wards of general hospitals). We analyzed the differences between the mean scores setting a significance level of 95%.

The open-ended questions were assessed using content analysis [28]. We calculated the percent frequencies of the topics most frequently mentioned by nurses.

This research was carried out in compliance with the Helsinki Declaration. The Ethics Commission of the Italian Society of Pediatric Nursing Sciences (SISIP) gave its consent to the study.

## Results

We collected 536 questionnaires. 27.2% (n = 146) of the nurses who filled out the questionnaires worked in a pediatric hospital and 59.3% (n = 318) worked in the pediatric ward of a general hospital, while 13.4% (n = 72) of respondents did not specify their place of work.

70.7% (n = 379) of the nurses who filled out the questionnaires worked in hospitals located in northern Italy, with the majority of them (33.3%, n = 126) coming from Lombardy. 17.3% (n = 93) of respondents worked in hospitals located in central Italy, with the majority of them (63.4%, n = 59) coming from Tuscany. Finally, 11% (n = 42) of respondents worked in hospitals located in southern Italy, with the majority of them (52.4%, n = 22) coming from the Campania region.

Table 2 shows the distribution of answers to the 10 closed-ended questions.

**Table 2:** **Synoptic table of the replies given by the nurses who filled out the questionnaires (n = 536)**

**Question**	**Mean value**	**1**	**2**	**3**	**4**	**5**
		**never**	**rarely**	**sometimes**	**often**	**always**
		**%**	**%**	**%**	**%**	**%**
A) Are children allowed to have their parents (or parent substitute) with them at all times?	4.47	1.5	4.4	9.1	15	70
B) Do children have the possibility of playing freely and of having their toys with them?	4.29	1.3	6.1	11.9	23.5	57.2
C) Do children and their parents receive information on their condition and on medical procedures?	3.94	0.9	7.8	19.4	39.3	32.6
D) Are children’s parents involved and encouraged to actively participate in their health care?	4.08	0.8	5.1	17.6	37.9	38.6
E) Are children encouraged to express their informed assent to/dissent with the diagnostic and therapeutic decisions involving them?	3.01	12.4	21.7	29.5	24.7	11.7
F) Are parents offered proper and comfortable accommodation in order to allow them to stay with their children during the whole day?	3.37	3.8	22.4	28.5	23.3	22.0
G) Are children cared for together with other children who have the same developmental characteristics?	3.39	7.3	13.1	30.3	31.7	17.9
H) Is children’s privacy respected at all times and by all people caring for them?	3.74	1.3	12.0	24.1	35.6	27.0
I) Are children offered the possibility of continuing their school education?	3.07	20.2	17.1	17.3	25.5	19.8
J) Are all precautions taken and all available means used in order not to cause pain to children during invasive procedures?	3.41	2.5	14.0	36.5	34.0	13.0

The most frequently implemented right was reported to be the right for children to have their parents with them at all times (85% answered "often" or "always"). However, in contrast to this data, the right to a proper and comfortable accommodation for parents in order to allow them to stay with their children during the whole day is implemented much less frequently (45.3% answered "often" or "always"). The right of children to play and have their toys with them is also very frequently implemented (80.7% answered "often" or "always") while their right to continue their school education is one of the least frequently respected rights (45.3% answered "often" or "always"). The right that was reported to be least respected is the right of the child to express their informed assent or dissent with regard to decisions that involve them (36.3% answered "often" or "always"); on the other hand, children’s parents are involved and encouraged to actively participate in health care "often" or "always" according to 76.5% of the respondents. Disappointingly, the right not to feel pain during invasive procedures is guaranteed "often" or "always" according to 47% of the respondents, but "always" only in 13% of reports.

With respect to the open-ended question on the most neglected children’s right, the majority of nurses replied that it is the right to pain prevention and treatment (16%, n = 86). Secondly, they indicated the children’s right to express their opinions and, in particular, their assent/dissent with regard to the diagnostic and therapeutic procedures they have to undergo (9.3%, n = 50).

Other neglected rights were the right to play and recreation (7.6%, n = 41), the right to receive information on the therapeutic process (7.2%, n = 39) and the right to privacy (5.2%, n = 28).

Another right which was considered to be neglected by 1.6% of respondents (n = 9) was the right to a child-friendly environment.

With respect to the open-ended question on the most important rights, respondents mentioned most of all the children’s right to have a parent, or parent substitute, with them at all times (20.3%, n = 109), followed by the need to take all possible measures to limit and prevent pain (15.2%, n = 82) and by the children’s right to have their psycho-physical wellbeing respected at all times (7.4%, n = 40). 6.3% (n = 34) of respondents stated that the most important right is the right to receive the best possible care by staff adequately trained in the pediatric field; 5.2% (n = 28) of respondents mentioned the right to play; 4.2% (n = 23) indicated the children’s right to receive information on the health care procedures they have to undergo. Finally, 2.6% of respondents replied that the most important right is the right to a reassuring and child-friendly environment.

Table 3 shows the differences in mean scores between respondents working in northern and central Italy and respondents working in southern Italy for each question.

**Table 3:** **Differences in mean scores between respondents working in different areas of Italy for each question (n = 536)**

**Question**	**Mean north/central**	**Mean south**	P*
A) Are children allowed to have their parents (or parent substitute) with them at all times?	4.502	4.208	0.039
B) Do children have the possibility of playing freely and of having their toys with them?	4.313	4.085	ns
C) Do children and their parents receive information on their condition and on medical procedures?	4.150	3.50	0.00001
D) Are children’s parents involved and encouraged to actively participate in their health care?	3.99	3.64	0.015
E) Are children encouraged to express their informed assent to/dissent with the diagnostic and therapeutic decisions involving them?	3.05	2.74	ns
F) Are parents offered proper and comfortable accommodation in order to allow them to stay with their children during the whole day?	3.40	3.02	0.03
G) Are children cared for together with other children who have the same developmental characteristics?	3.41	3.26	ns
H) Is children’s privacy respected at all times and by all people caring for them?	3.77	3.44	0.034
I) Are children offered the possibility of continuing their school education?	3.45	2.93	0.004
L) Are all precautions taken and all available means used in order not to cause pain to children during invasive procedures?	3.77	3.44	0.034

* Anova test.

All the children's rights are reportedly more frequently implemented in northern and central Italy than in southern regions, with the exception of the right to play and have their toys, the right to express their assent or dissent and the right to be taken care of with children of the same developmental age.

Table 4 shows the differences in mean scores between respondents working in pediatric hospitals and respondents working in the pediatric wards of general hospitals for each question.

**Table 4:** **Differences in mean scores between respondents working in pediatric and in non-pediatric hospitals (n = 536)**

**Question**	**Pediatric ward of a general hospital**	**Pediatric hospitals**	P*
A) Are children allowed to have their parents (or parent substitute) with them at all times?	4.51	4.37	ns
B) Do children have the possibility of playing freely and of having their toys with them?	4.35	4.11	0.014
C) Do children and their parents receive information on their condition and on medical procedures?	4.11	4.03	ns
D) Are children’s parents involved and encouraged to actively participate in their health care?	3.97	3.86	ns
E) Are children encouraged to express their informed assent to/dissent with the diagnostic and therapeutic decisions involving them?	2.96	3.14	ns
F) Are parents offered proper and comfortable accommodation in order to allow them to stay with their children during the whole day?	3.46	3.12	0.0029
G) Are children cared for together with other children who have the same developmental characteristics?	3.39	3.41	ns
H) Is children’s privacy respected at all times and by all people caring for them?	3.78	3.64	ns
I) Are children offered the possibility of continuing their school education?	2.84	3.69	0.00001
L) Are all precautions taken and all available means used in order not to cause pain to children during invasive procedures?	3.40	3.42	ns

* Anova test.

The right of children to play and the right to have their parents comfortably accommodated are reported to be significantly more respected in pediatric units of general hospitals than in pediatric hospitals, while the right to school education is reported to be significantly more guaranteed in pediatric hospitals than in general hospitals.

## Discussion

This is the first study describing to which extent the rights of sick children are respected in Italian pediatric hospitals and wards and, as compared to similar studies conducted in other countries, is the one with the largest number of participants published so far [2,26].

Our results show that there is a considerable variability in the implementation of children's rights as perceived by nurses: while some rights seem widely respected (right to have a parent with them, right to play and to have toys), others have much lower levels of implementation (right to express informed assent or dissent, right to continue school).

Also, it is interesting to note that the limited implementation of some rights has the potential to reduce the positive effects of other rights that are implemented more widely: for instance, although the right of children to have their parents close to them is generally guaranteed (85% replied "often" or "always"), the implementation of the right to a proper and comfortable accommodation for parents is considerably more limited (45.3% responded "often" or "always"), thus making the stay of parents at their child's bedside potentially difficult.

Another aspect that emerges from our study is that almost all the children's rights are more frequently implemented in northern and central Italy than in southern regions; this finding is consistent with recent reports describing significant inequalities between northern and southern Italy in the health care services provided by regional health systems [29].

The most important hospital accreditation agencies generally provide accreditation standards for pain management, including adequate assessment and treatment [30]. Similar requirements are also provided by an Italian Law, Act 38 of 2010 [31]. It is therefore disappointing to note that according to our study the right not to feel pain is respected "often" or "always" in Italian pediatric units only in less than half of cases. This is consistent with the answers to the first open-ended question, where the majority of nurses reported that the right to pain prevention and treatment is the most neglected children’s right.

The United Nations Convention on the Rights of the Child, which has been an integral part of the Italian legislation since 1991, establishes the right of children to "express their views freely in all matters affecting them" [32]. In contrast with this provision, our data show that children’s right to express their opinions and, in particular, their assent/dissent with regard to diagnostic and therapeutic procedures is the least respected right in Italian pediatric hospitals.

Only two other studies investigating the actual implementation of children's rights in hospital have been published to date [2,26]; although the methods used are different, it is possible to compare some of their results with ours. In the study by Migone et al. carried out in Ireland [2], 50% of nurses stated that children are encouraged to ask questions; this figure is higher than that resulting from our study, where, according to nurses, children are encouraged to express themselves "often" or "always" in 36.4% of cases. Also, in the Irish study adequate access to play and to education was reported by nurses in 36% and 40% of cases, respectively, whereas in our study it was guaranteed "often" or "always" in 80.7% and 45.3% of cases, respectively. However, this comparison should be interpreted with caution, due to the methodological differences between the studies.

With regard to the survey by the International Network of Health Promoting Hospitals and Health Services [26], the right to play was reported as "significantly achieved" in 57.1% of participating hospitals and the right of children to express their opinion in 7.1%; moreover, the right not to be separated from parents was "significantly achieved" in 53.3% of hospitals (85% "often" or "always" in our study) and the right not to feel pain in 46.7% (47% "often" or "always" in our study).

A possible limitation of our study may be represented by the indirect collection of information, i.e. by means of the opinions of nurses who work in the various pediatric settings. This limit is common to the other two studies on the same subject. The results would have certainly been more informative if we had collected the opinions of users or if we had used validated assessment tools based on objective parameters – which currently do not exist.

Another possible limitation consists of the way subjects were invited to participate in the study and how they answered the questionnaire. One may speculate that spreading and distributing the questionnaire on paper during pediatric nursing conferences and by means of the Internet and social networks may have somehow selected those nurses more prone to keeping themselves up to date and to using new information technologies as participants, and this may have influenced the results. Also, we are not able to estimate how many pediatric nurses who received the invitation to participate through online social networks may have decided not to answer the questionnaire. However, since the total number of pediatric nurses in Italy is approximately 11,000 [33], we can estimate that we included about 4.9 % of Italian pediatric nurses in the study.

## Conclusion

Our study shows that the implementation of the rights of hospitalized children in Italian pediatric units is still limited and not uniform. A scarce implementation of the rights of sick children in the actual clinical and organizational practice of pediatric units may lead to negative consequences on the quality of life of hospitalized children and their family. However, this study allowed us to provide data – which were previously not available – that can help decision-makers to take measures to improve the implementation of some rights of children hospitalized in Italian pediatric hospitals and pediatric wards of general hospitals.
